# Authors reply: comments on the published meta-analysis of antibiotic resistance in hospital-acquired ESKAPE-E infections in low- and lower-middle-income countries

**DOI:** 10.1080/22221751.2022.2122586

**Published:** 2022-09-27

**Authors:** Olaniyi Ayobami, Simon Brinkwirth, Tim Eckmanns, Robby Markwart

**Affiliations:** a Unit for Healthcare Associated Infections, Surveillance of Antimicrobial Resistance and Consumption, Department of Infectious Disease Epidemiology, Robert Koch Institute Berlin, Germany; b Jena University Hospital, Institute of General Practice and Family Medicine Jena, Germany

**Keywords:** Antibiotic resistance, Hospital-acquired infection, Low-resourced countries, Health equity, ESKAPE-E pathogens

We thank Hadi and colleagues[1] for the interesting comments on our study[2] and we are happy to provide replies to the specific comments raised.

First, Hadi and colleagues[1] stated we did not use the quality assessment in a subgroup analysis to perform investigate the sources of heterogeneity. We agree that conducting a subgroup analysis in meta-analysis is important to investigate heterogenous results or answer specific questions. While we assessed for risk of bias on three criteria (sample selection, microbiology method, and representativeness), the regional or national representativeness of the studied hospital population was an important criterion for assessing the validity of the estimates since this study was conducted to provide regional proxy estimates for L-LMIC regions in the absence of fully functional national and regionally-representative antimicrobial resistance (AMR) surveillance systems. The overall risk of bias in the majority of the studies is moderate or high (162/163, 99.4%) and only one study was deemed to have a low risk of bias. However, when analyzed per criterion, the risk of bias for the regional or national representativeness of the studied hospital population was assessed as “high” in virtually all studies (n = 161/163, 98.8%). Therefore, based on this unsurprising finding and acknowledged limitation, we do not consider our overall risk of bias to be of intrinsic value to categorize a sufficient number of studies as “high or low quality” for a meta-regression. Nevertheless, in order to reliably explore possible sources of observed heterogeneity while reducing false-positive associations, we assessed for systematic differences in our outcome with a moderator analysis (meta-regression) including study characteristics such as whether the study is multicenter or single center, WHO region, Income level, Age group, and HAI type as pre-specified in the protocol. These findings reiterate the need for caution in the interpretation of pooled estimates and the urgent need for an expanded AMR surveillance capacity in L-LMICs regions that provides more reliable estimates by reducing selection bias that a meta-analysis of published studies may not adequately exclude.

Secondly, Hadi and colleagues raised concerns about the importance of perform subgroup analysis based on the guideline or the breakpoints used to interpret the AST results to ensure similar definitions of antibiotic resistance. For this study, we extracted data on AST guidelines used to determine included studies that rely on validated guidelines and are not as a part of our pre-specified subgroup analysis per protocol. In total, 77.9% (127/163) of the included studies reported the guideline used to interpret the AST results. In 36 of the included studies, no guideline was reported*.* Almost all of the included studies with reported AST guideline used AST interpretations based on the CLSI (116/127, 91.3%). In three studies, the interpretation was based on EUCAST, and another eight studies used different guidelines than these. Even though different breakpoints within the interpretation guidelines can cause heterogeneity in the study results, the subgroups with 3 studies using EUCAST compared to 127 using CLSI guidelines would not be adequate to reliably conduct and interpret a subgroup analysis. Also, investigations of heterogeneity when there are very few studies are of questionable value [3]. In the discussion of the study findings, we noted that the methodological differences in AST as a limitation that might explain the high heterogeneity in our main discussion, especially when comparing our study results within L-LMICs and between different national surveillance data (Table 1). The AST guidelines of most included surveillance data were based on CLSI (USA, ReLAVRA, Japan, and China), while surveillance data from EU/EEA and Germany were primarily based on EUCAST. Therefore, direct comparison between these groups is restated to be done with caution. Still, we acknowledge that meta-analyses directly based on the breakpoints used to interpret AST results will be a more accurate approach. However, the lack of breakpoint reporting in many studies, the variability of methods used, and the changes in breakpoints within the same guideline such as CLSI over the period of the included studies (2010-2020) make such analysis difficult, especially with insufficient details in published observational studies.

**Table 1.** Analysis of publication bias.

Pathogen	Resistance	Number of studies	Egger’s test* [12]	Begg’s test [13]
*E. coli*	Carbapenems	60	t = −1.65, df = 58, *p*-value = 0.1039	z = 1.24, *p*-value = 0.2155
*K. pneumoniae*		50	t = −0.44, df = 48, *p*-value = 0.6638	z = −0.26, *p*-value = 0.7952
*P. aeruginosa*		56	t = −1.25, df = 54, *p*-value = 0.2177	z = −1.19, *p*-value = 0.2348
*Enterobacter* spp.		7	-**	-**
*A. baumannii (complex)*		36	t = −2.40, df = 34, *p*-value = 0.0221	z = −2.49, *p*-value = 0.0126
*E. coli*	Third-generation cephalosporins	58	t = −1.65, df = 58, *p*-value = 0.1039	z = 1.24, *p*-value = 0.2155
*K. pneumoniae*		48	t = −1.19, df = 44, *p*-value = 0.2401	z = −0.74, *p*-value = 0.4595
*Enterobacter* spp.		8	-**	-**
*S. aureus*	Methicillin	80	t = 0.47, df = 78, *p*-value = 0.6365	z = 0.61, *p*-value = 0.5411
	Vancomycin	39	t = 2.21, df = 37, *p*-value = 0.0337***	z = 4.92, *p*-value < 0.0001***

* Predictor: standard error, weight: inverse variance

** Number of studies too small to test for small study effects (k_min_ = 10)

*** The majority of studies reported proportions of 0 or close to 0. As stated above, funnel plots were found to be an inaccurate method of assessing publication bias in proportion studies with low proportion outcomes [8].

Lastly, Hadi and colleagues raised concerns about the absence of any investigation of publication bias in our study. Publication bias has been long recognized as a serious problem in clinical research [4–6]. There is clear evidence that studies are more likely to be published if their results are statistically significant, or confirm the initial hypothesis, such as the clinical effectiveness of the investigated drug or clinical intervention [7–9]. Therefore, in meta-analyses, the publication bias can be analyzed using different methods, such as assessing the asymmetry of funnel plots quantified by Egger's regression test and Begg's rank test. However, in the original manuscript, we decided to not perform publication bias analyses based on two main reasons:

1. To our knowledge, in contrast to clinical effectiveness studies (either RCT or non-RCTs), there are no systematic data on the importance and extent of publication bias in prevalence/incidence studies like ours. The authors admittedly have not reached a consensus on the nature of publication bias in a prevalence/proportion meta-analysis. Likewise, as shown by Migliavaca et al. 2020 [10] publication bias was examined in less than half of systematic reviews of prevalence studies.
2. It appears that there is no accepted standard on a robust quantitative method to analyse publication bias in proportion studies. The reliability of the conventional funnel plots in assessing publication bias in meta-analyses of proportion studies remains questionable, especially in proportion studies with low proportion outcomes [11].

However, in response to the commentary, we conducted publication bias analyses in our main analyses. We were able to detect funnel plot asymmetry in only two out ten analyses (see table below). Importantly, the funnel plot asymmetry detected in the analysis of vancomycin resistance in *S. aureus* may not be accurate, since the majority of these studies reported proportions of zero or close to zero. In sum, based on our funnel plot analyses, we conclude that publication bias (small-study effects) is not prominently present in our meta-analyses.
